# Proteomic Biomarkers as Mediators in the Association Between Air Pollution and Incident Depression and Anxiety: Evidence From the UK Biobank

**DOI:** 10.1155/da/3984517

**Published:** 2025-06-26

**Authors:** Mingqia Wang, Yu Ma, Yue Zheng, Yanbao Tao, Wanrong Mu, Liu Yang, Jiayi Li, Zihan Li, Chuan Shi

**Affiliations:** ^1^Peking University Sixth Hospital, Peking University Institute of Mental Health, NHC Key Laboratory of Mental Health (Peking University), National Clinical Research Center for Mental Disorders (Peking University Sixth Hospital), Beijing, China; ^2^Suzhou Guangji Hospital, Suzhou, Jiangsu, China; ^3^Department of Psychiatry, Xinxiang Medical University, Xinxiang, China; ^4^Department of Psychiatry, The Affiliated Brain Hospital of Guangzhou Medical University, Guangzhou, China

## Abstract

**Background:** The association between ambient air pollution and the onset of depression and anxiety has attracted significant scholarly interest; however, its underlying mechanisms remain elusive.

**Objective:** The primary objective of this study is to assess the connection between air pollution and the onset of depression and anxiety, with a specific emphasis on uncovering the potential pathways influenced by high-dimensional proteomic markers.

Design, setting, and participants: This prospective, nationwide population-based cohort study utilized data from the UK Biobank, incorporating an analysis of proteomic data from 38,301 participants.

**Method:** Air pollution levels for particulate matter (PM) diameters of ≤2.5 μm (PM_2.5_), ≤10 μm (PM_10_), 2.5–10 μm (PM_coarse_), nitrogen oxides (NO_x_), and nitrogen dioxide (NO_2_) were estimated in 2010. The depression and anxiety symptoms were assessed through self-report questionnaires at baseline and at the 7-year follow-up, and diagnoses were determined using ICD-9/10 codes from hospital records. Plasma proteomic data for 1463 proteins was measured using the Olink platform. A two-step regression approach was used to identify proteins associated with both air pollution and depression/anxiety. Mediation analysis was performed using the med4way method.

**Results:** This longitudinal nationwide study leveraged the UK Biobank cohort to elucidate the protein-mediated effects of five major air pollutants on depression and anxiety. The findings identified 23 proteins mediating the risk of developing depression associated with air pollution, while no mediating proteins were found for anxiety. Additionally, the study discovered 38 proteins linked to the severity of anxiety related to air pollution and nine proteins connected to the severity of depressive symptoms. Notably, KEGG pathway analysis revealed significant associations with critical signaling cascades, such as the estrogen, IL-17, and pathways. Furthermore, STRING analysis underscored the shared roles of specific proteins, including EGFR, IL15, CCL2, and CCL20, in the context of air pollution-induced depression and anxiety, highlighting the involvement of immune-related processes and pathways.

**Conclusion:** The findings of this large population-based cohort study provide proteomic evidence on the mediating protein associations between air pollution and the onset of depression. The results suggest that the immune system plays a significant role in the biological mechanisms linking air pollution to depression and anxiety.

## 1. Introduction

Globally, 322 million people are living with major depressive disorder (MDD), while 264 million are affected by anxiety disorders (AD), both of which are common mental health issues worldwide [1]. These disorders, which often co-occur, have profound implications for individuals' quality of life and overall societal well-being. A full understanding about the modifiable risk factors of MDD and AD is of great importance to public health [2].

Air pollution, as a modifiable risk factor, characterized by the presence of harmful substances like particulate matter (PM) and nitrogen oxides (NOx), poses a significant environmental health risk. In 2019, 99% of the global populace inhabited regions where the air quality standards set by the World Health Organization were not satisfied [3]. Numerous studies have demonstrated that air pollution, is linked to a wide array of adverse health outcomes, with recent years seeing growing attention directed towards its impact on mental health [4]. Prior research on the link between air pollutants and depression/anxiety mainly focused on individual air particles [5, 6]. However, air pollution is a complicated combination of PM and gaseous pollutants, and its general health effects may vary considerably from those of specific pollutants. In response, scientists developed an expanded analysis technique called Air Pollution Indexes. These indices use demographic impact estimates to weigh pollutants, giving a more systematic assessment framework that prioritizes components with stronger associations to the diseases, as opposed to traditional single-pollutant analysis metrics [7].

Epidemiological evidence has increasingly affirmed the association between air pollution exposure and the heightened risk of mental health disorders, particularly depression and anxiety [4, 8]. Recent research utilizing data from the UK Biobank has highlighted the significant impact of long-term exposure to air pollutants on mental health, specifically depression and anxiety. Yang et al. [5] conducted a comprehensive cohort study that linked prolonged exposure to PM with a diameter of ≤2.5 μm (PM_2.5_) and/or ≤10 μm (PM_10_) and NO_x_ (NO_2_ and NO) with increased risks of incident depression and anxiety. Their findings revealed a nonlinear exposure-response relationship, characterized by a steeper slope at lower exposure levels and a leveling off at higher concentrations. Similarly, Gao et al. [9] demonstrated that elevated levels of PM_2.5_, PM_10_, NO_x_, and NO_2_ were associated with higher odds of incident depression and anxiety, particularly among individuals with a genetic predisposition to these mental disorders. These studies underscore the importance of understanding environmental factors, such as air pollution, in the context of depression and anxiety.

Proteomics has emerged as a crucial tool for elucidating the complex mechanisms linking air pollution with depression and anxiety, providing significant insights into the pathogenic processes involved. Research has shown that exposure to pollutants such as PM_2.5_, NO_2_, and CO can substantially modify human proteomic profiles, revealing pathways associated with oxidative stress [10, 11] and inflammation [12, 13]. Furthermore, proteomic analyses are vital for predicting disease risk, with recent studies utilizing UK Biobank data identifying a proteomic risk score linked to depression and anxiety, thereby emphasizing the potential of plasma protein profiles in evaluating mental health risk [14]. Population-based research into the pathways between air pollution and mental disorders constitutes a complex field, involving various biological, environmental, and societal factors [15, 16]. Mediation analysis using proteomics offers a method to delve into these relationships, aiding in our understanding of the mechanisms underlying these intricate links [17]. Despite these developments, there is still only so much research in this field. Earlier studies have mostly focused on air pollution exposure, which is likely to significantly impact mental health, which includes in-depth examinations of the modification factor between air pollutants and mental health, but without any investigation into potential molecular mediating factors [9, 18, 19]. We hope this research will advance our understanding of the biological mechanisms underlying the air-pollution-depression/anxiety relationship and provide information from a large-scale population-based observational study, given that many studies on biological mechanisms are derived from animal experiments.

Traditional mediation techniques frequently fail to consider the possible interplay between exposure-mediator interactions and mediation effect which prevents them from identifying whether observed associations result from direct biological processes (mediation), synergistic effects (interaction), or a combination [20]. The med4way decomposition method [21] was used to address this. By simultaneously quantifying both mediation and interaction effects, this cutting-edge causal-inference framework is very useful for uncovering the novel mechanisms found in environmental epidemiology. It also effectively overcomes the main limitations of conventional approaches. There haven't been any studies to date that compare long-term exposure to air pollution, proteomics, and depression/anxiety in population-based settings. Therefore, it is imperative to explore the linkage between air pollution and depression through a comprehensive and prospective cohort study. In this retrospective cohort study, we are utilizing data from the UK Biobank to assess the mediation of depression and anxiety linked to air pollution through plasma proteomics. Our primary objective is to identify significant mediator proteins associated with these emotional conditions. Additionally, we integrated proteomics data into a Cox proportional hazards regression mediation model to establish individual risk models for the onset of depression and anxiety induced by air pollution, and to investigate proteins that may mediate these effects.

## 2. Methods

### 2.1. Study Design and Participants Selection

**Figure 1:**
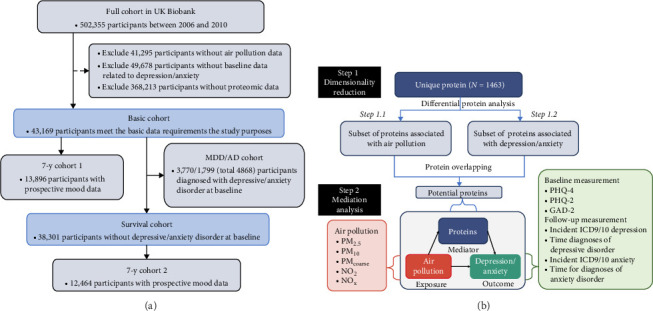
Study design and participants selection. **(A)** Study design overview in this study. **(B)** Participant selection flowchart for integration of air pollution, depression, and proteomic data.

The study design and participants from the UK Biobank have been extensively documented previously [22]. The complete cohort of the UK Biobank comprises 502,355 participants recruited between 2006 and 2010, aged 37–73 years at baseline, undergoing multiple follow-up assessments in an ongoing prospective study. At baseline, participants were recruited from 22 medical health centers across the UK, where they provided lifestyle and health information and biological samples. These biological samples were utilized for various quantitative assessments of biomarkers, including proteomic analysis. The selection process of participants is delineated in Figure 1A, while the study design overview of this investigation is depicted in Figure 1B.

### 2.2. Air Pollution Exposure

In 2010, the UK Biobank utilized the land use regression (LUR) model, developed as part of the European study of cohorts for air pollution effects (ESCAPE), to calculate the annual average environmental concentrations of PM_2.5_, PM_coarse_, PM_10_, NO_2_, and NO_x_ [23, 24]. This model leveraged geographic information systems (GIS) to derive predictor variables like land use, traffic, and topography at a 100 m × 100 m resolution. The estimation data for PM was considered reliable within a 400-km radius of the Greater London monitoring zone, where data beyond this distance was deemed missing. Consequently, 41,295 individuals, representing a 8.2% missing rate, were excluded due to their missing air pollution data. The Air Pollution Index, a continuous weighted score reflecting the combined concentrations of air pollutants adjusted by their impact estimates on the odds of MDD/AD at baseline or the risk of MDD/AD at the follow-up, was calculated as follows: Air Pollution Index = (*β* [PM_2.5_] × PM_2.5_ + *β* [PM_10_] × PM_10_ + *β* [NO_2_] × NO_2_ + *β* [NO_x_] × NO_x_) × (4/sum of the *β* coefficients), as a higher score indicating higher exposure to ambient air pollution [7].

### 2.3. Depression and Anxiety Assessment

At baseline and follow-up, depression/anxiety was assessed using relevant hospital records and mental health questionnaires. Regarding hospital records, participants with primary or secondary diagnoses of International Classification of Diseases-9/10 depression (ICD-9:311; ICD-10: F32-F33) and/or anxiety (ICD9:300; ICD-10: F40-F41) were classified as cases. At baseline, depression and anxiety symptoms were assessed using the Patient Health Questionnaire (PHQ)−4 [25], which screens for both conditions by combining the PHQ-2 and GAD-2. In the 7th-year assessment, depression was measured utilizing the PHQ-9 scale, with a score of ≥ 10 indicating positive depression status [26], while anxiety was assessed through the 7-item Generalized Anxiety Disorder scale (GAD-7), with a cutoff score of ≥ 8 indicating positive anxiety status [27]. Participants were asked to rate the questionnaires on a 4-point Likert scale from 0 (not at all) to 3 (nearly every day).

### 2.4. Proteomic Assays

Blood-based proteomic data were generated by the UK Biobank Pharma Proteomics Project Consortium. From April 2021 to January 2022, these plasma samples were sent to the Olink analysis service center in Sweden, where 1463 unique proteins from four panels (representing cardiovascular metabolism, inflammation, neurology, and oncology) were measured using proximity extension assay conducted by Olink [28]. Detailed information regarding sample selection, processing, data normalization, and stringent quality control has been thoroughly disclosed at https://biobank.ndph.ox.ac.uk/ukb/ukb/docs/Olink_proteomics_data.pdf. In our study, we ultimately included 1463 unique proteins, utilizing standardized protein expression (NPX) values as the metric for relative protein quantification for subsequent proteomic analysis. The full names and abbreviations of these proteins relevant to this study can be found in the Appendix.

### 2.5. Measurement of Covariates

To address potential biases associated with factors related to mental health and air pollution [9, 29–31], we included the following baseline data: age (years; continuous), gender (male/female; categorical), body mass index (BMI, kg/m^2^; continuous), ethnicity (Asian/Black/Multiracial/other/White; categorical), education level (college or university/other; categorical), proximity to major roads (in 1km, continuous), greenspace (within 1000 m buffer, continuous), duration of residency (years; continuous), household income (Less than 18,000/18,000 to 30,999/31,000 to 51,999/52,000 to 100,000/greater than 100,000, ordinal categorical), Townsend deprivation index (continuous), healthy alcohol intake status (yes/no; categorical), healthy physical activity status (yes/no; categorical), educational qualifications (college or university degree/other; categorical), forced expiratory volume in the first second (FEV1, liters; continuous), forced vital capacity (FVC), (liters; continuous), urban residency (yes/no; categorical), and the presence of diabetes, hypertension and coronary heart disease (CHD) (yes/no; categorical) prior to recruitment.

BMI was determined by dividing weight by height squared, based on measurements taken by trained nurses during the initial assessment. Healthy alcohol intake was defined as less than 28 g per day for males and less than 14 g per day for females [32]. Healthy physical activity was defined as engaging in at least 150 min of moderate exercise, 75 min of vigorous exercise, or a combination of both totaling 150 min per week, as assessed by the International Physical Activity Questionnaire (IPAQ) [32, 33]. The Townsend deprivation index is a commonly used measure in social science and public health research to evaluate the level of deprivation within a community or geographical area, with a higher index indicating greater deprivation. Histories of hypertension, CHD [34], and diabetes [35] were obtained through self-reported information and medical records.

### 2.6. Statistical Analysis

We used a two-step process to create a powerful mediation model, using Cox proportional hazards regression for survival outcomes and linear models for continuous outcomes. Initially, we utilized the Air Pollution Index as the primary predictor in a series of separate multiple linear regression models, treating each of the 1463 plasma proteomic biomarkers as the outcome of interest, while simultaneously adjusting for the aforementioned covariates. Secondly, each of the 1463 plasma proteomic biomarkers was treated as the main predictor in a series of separate multiple linear regression models, with the six outcome measures (diagnoses of ICD-9/10 depression, diagnoses of ICD-9/10 anxiety, PHQ-2, GAD-2, PHQ-9, and GAD-7) being alternated as the outcomes of interest, again adjusting for the same covariates mentioned above. Subsequent to the regression analyses, we applied a false discovery rate (FDR) correction to identify proteins that were significantly associated with both air pollution and depressive/anxiety symptoms. The intersection of these differentially expressed proteins was then determined, representing the set of proteins potentially mediating the relationship between air pollution and MDD/AD. Volcano plots were generated using the *R* ggplot package to illustrate the *p*-values and estimate of each of the 1463 equations and to highlight those that passed multiple testing with the FDR correction. Furthermore, Venn diagrams were used to show the overlap of different proteins, highlighting shared, unique, and nondifferential categories.

The med4way method is applicable in various scenarios: when the outcome variable can take the form of a continuous variable, a dichotomous variable or a survival time variable, and the mediator variable can be either continuous or binary. In this study, it was utilized to construct models that link the Air Pollution Index to selected shared proteins and subsequently to MDD/AD outcomes. This method decomposes the overall effect of an exposure on an outcome by considering potential interactions with a mediator. The decomposition includes the following components: the effect due to neither mediation nor interaction, the effect due to interaction alone (without mediation), the effect due to both mediation and interaction, and the effect due to mediation alone (without interaction) [21]. The findings from the med4way analysis are categorized as follows: (A) no mediation was observed pure indirect effect (PIE) *p*  > 0.05); (B) inconsistent mediation was found when the controlled direct effect (CDE) attained statistical significance with its absolute value exceeding that of the total effect (TE) and demonstrating an opposing directionality to TE (PIE *p*  < 0.05, |CDE| > |TE|, and opposite sign to TE); (C) consistent mediation was identified (PIE *p*  < 0.05, |CDE| < |TE|, and same sign as TE) [36]. This comprehensive four-way decomposition enables a unified assessment of the contributions of both interactions and mediation effects to the overall association between exposures and outcomes. The exposures considered include individual measurements of PM_2.5_, PM_coarse_, PM_10_, NO_2_, and NO_x_. The outcomes include metrics such as PHQ-2, GAD-2, GAD-7 at 7-y, PHQ-9 at 7-y using linear regression model and hospital records related to depression and anxiety using Cox proportional hazards regression model. The main analysis was conducted in both the survival cohort and the 7-y cohort 2, excluding individuals already diagnosed with depression or anxiety at baseline. All analyses were performed using R 4.3.2 and Stata 18.

To elucidate the primary biological functions and signaling pathways involved in proteins showing consistent mediation, we conducted gene enrichment analysis. We employed gene ontology (GO) analysis method and utilized Fisher's exact test to assess statistical significance. Concurrently, Kyoto Encyclopedia of Genes and Genomes (KEGG) enrichment analysis, protein–protein interaction (PPI) analysis were performed aiming to investigate potential mechanisms.

For the sensitivity analysis, we conducted parallel studies using UK Biobank data: one excluding participants with a documented history of depression and another including those diagnosed with depression and/or anxiety using Basic cohort, and 7-y cohort1, including individuals diagnosed with depression and/or anxiety from the Basic cohort and the 7-year cohort. We employed linear modeling for dependent variables PHQ-2, GAD-2, PHQ-9 at 7-y, and GAD-7 at 7-y, utilizing their respective cutoff values. Additionally, for the med4way survival analysis model, logistic regression was employed. We compared the results of logistic regression with those of linear modeling.

## 3. Results

### 3.1. Participants' Characteristics

This study includes a total of 38,301 subjects with comprehensive data on air pollution, depression/anxiety, and proteomics, excluding participants with MDD and AD.  Table 1 presents demographic and environmental characteristics of the study population filtered based on three sequential screening steps. Our primary analysis focuses on the survival cohort. The survival cohort subset has a mean age of 56.93 years with a standard deviation of 8.22 years. The distribution across age groups is well-balanced, and females constitute 52.2% of the sample. Regarding race and ethnicity, the majority identify as White (94.0%), with smaller percentages identifying as Asian, Black, Multiracial, and other ethnicities. Detailed documentation of environmental exposures includes proximity to major roads, ambient noise levels, availability of greenspace, and concentrations of air pollutants (e.g., NO_2_, NO_x_, PM_10_, PM_2.5_, and PMcoarse see Table 1), revealing significant correlations.

### 3.2. Air Pollution Index

According to Wang et al. [37], we computed two Air Pollution Indexes—a continuous weighted score reflecting the combined concentrations of key air pollutants adjusted by their impact estimates on the odds of MDD/AD at baseline. Specifically, these indexes are the MDD-related Air Pollution Index and the AD-related Air Pollution Index. To examine the relationship between the MDD-related/AD-related Air Pollution Index and five major pollutants, we conducted a correlation analysis, which revealed the associations between the generated Air Pollution Index and its components (Supporting Information 1: Figure S1). The results indicate that the MDD-related Air Pollution Index exhibits correlation coefficients greater than 0.8 with PM_2.5_, NO_x_, and NO_2_, while showing lower coefficients with PM_10_ and PM_coarse_. Similar results were observed between AD-related Air Pollution Index and five major pollutants.

### 3.3. Differential Protein Analysis

**Figure 2:**
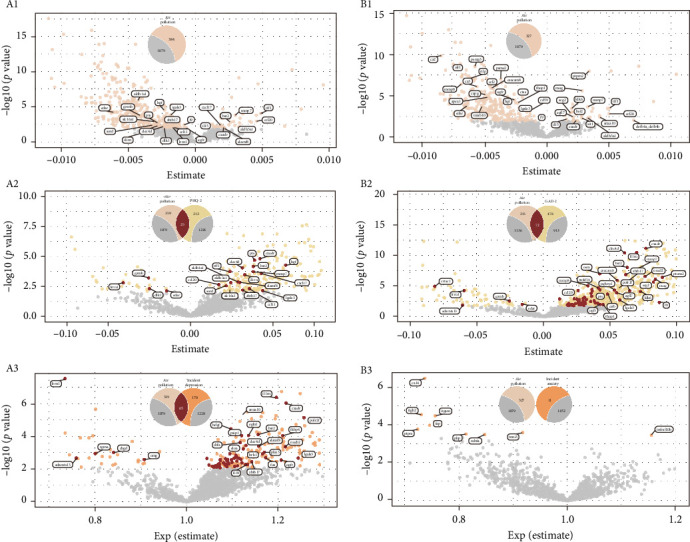
Volcano plots illustrating protein associations with various exposures and outcomes. (A1) Shows significant correlations between depression-related air pollution indices (adjusted for FDR) and 384 proteins. Red circles denote proteins with statistically significant associations, while gray circles represent proteins with no significant associations. (B1) Presents significant correlations between anxiety-related air pollution indices (adjusted for FDR) and 327 proteins. Red circles denote proteins with statistically significant associations, while gray circles represent proteins with no significant associations. (A2) Displays significant correlations between baseline PHQ-2 scores (adjusted for FDR) and 237 proteins. Among these, 25 proteins are significantly associated with both depression-related air pollution indices and baseline PHQ-2, marked with dark red circles. An additional 212 proteins are significantly associated only with baseline PHQ-2 and are shown with yellow circles. Gray circles indicate proteins with no significant associations. (B2) Shows significant correlations between baseline GAD-2 scores (adjusted for FDR) and 550 proteins. Of these, 72 proteins are significantly associated with both anxiety-related air pollution indices and baseline GAD-2, represented by dark red circles. The remaining 428 proteins are significantly associated only with baseline GAD-2 and are denoted by yellow circles. Gray circles represent proteins with no significant associations. (A3) Displays significant correlations between incident ICD9/10 depression (adjusted for FDR) and 235 proteins. Among these, 65 proteins are significantly associated with both depression-related air pollution indices and ICD9/10 depression, marked with dark red circles. An additional 170 proteins are significantly associated only with ICD9/10 depression and are shown with orange circles. Gray circles indicate proteins with no significant associations. (B3) Shows significant correlations between incident ICD9/10 anxiety (adjusted for FDR) and 11 proteins. These 11 proteins are significantly associated only with ICD9/10 anxiety, represented by orange circles. Gray circles denote proteins with no significant associations. The *x*-axis represents the beta coefficients from linear modeling of the variables of interest, while the *y*-axis represents -log10 (*p*-value). Venn diagrams illustrate the relationships between correlated proteins and the variables of interest.

In assessing the impact of depression-related air pollution indices on a proteomic dataset of 1463 proteins, 384 proteins demonstrated significant associations with the air pollution index after FDR adjustment (Figure 2 A1, Supporting Information 2: Table S1a). Analyzing correlations between baseline PHQ2 scores and protein levels identified 237 proteins with significant associations (Figure 2 A2, Supporting Information 3: Table S1b). Further scrutiny revealed that 25 (subset 1) of these 237 proteins also had significant correlations with the depression-derived Air Pollution Index (Figure 2 A1). When evaluating the association between depression onset risk and protein expression, 235 proteins were significantly correlated (Figure 2 A3, Supporting Information 4: Table S1c). Cross-referencing these with the 384 proteins linked to the depression-related air pollution index uncovered 65 (subset 2) shared proteins (Figure 2 A3). Regarding the longitudinal analysis of PHQ-9 scores over a 7-year period, 20 proteins exhibited significant associations (Supporting Information 5: Table S1d). Cross-referencing these with the 384 proteins related to the depression-derived air pollution index identified three overlapping proteins: A disintegrin and metalloproteinase with thrombospondin motifs 13(ADAMTS13), epidermal growth factor receptor (EGFR), and leucine-rich repeat neuronal protein 1 (LRRN1). Notably, EGFR and LRRN1 were consistently identified across all three groups of shared proteins.

In examining the influence of anxiety-related Air Pollution Index on the same proteomic dataset, 327 proteins were significantly associated with the air pollution index following FDR correction (Figure 2 B1, Supporting Information 6: Table S2a). Analysis of the relationship between baseline GAD-2 scores and protein levels revealed significant associations with 550 proteins (Figure 2 B1, Supporting Information 7: Table S2b). Further investigation showed that 72 (subset 3) of these 550 proteins also had significant associations with the anxiety-related air pollution index (Figure 2 B2). In the context of anxiety onset risk, 11 proteins were significantly correlated (Figure 2 B3, Supporting Information 8: Table S2c), however, none of these overlapped with those associated with the anxiety-derived air pollution index (Figure 2 B3, Supporting Information 8: Table S2c). Similarly, no proteins exhibited significant associations when analyzing GAD-7 scores over a 7-year follow-up (Supporting Information 9: Table S2d).

### 3.4. Mediation Analysis

**Figure 3:**
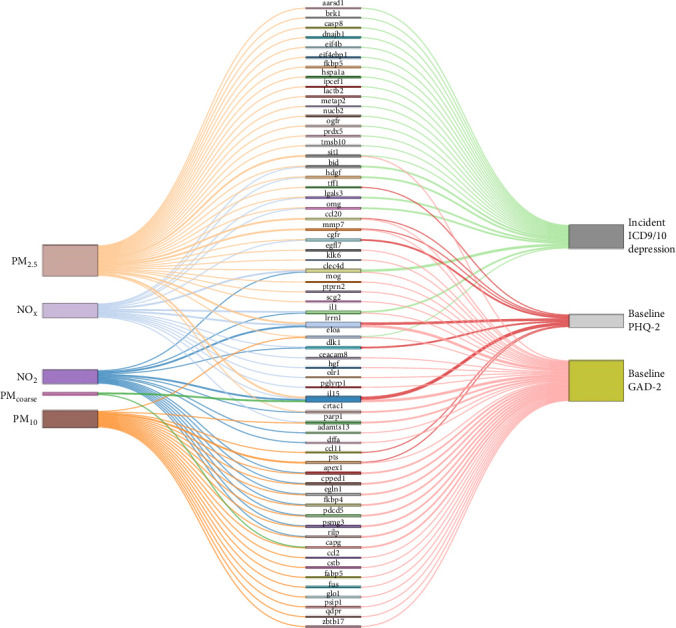
Sankey diagram illustrating the mediating effects of proteins on air pollutants and disease outcomes. The diagram visually represents the flow of associations between specific proteins, corresponding air pollutants, and disease outcomes, highlighting the mediating role of identified proteins. The width of the connections indicates the strength of the mediating effect, while the colors differentiate between various pollutants and disease outcomes. Full list of protein names presented in Figure 3 is provided below. LRRN1: Leucine rich repeat neuronal 1; DLK1: Delta like noncanonical notch ligand 1; IL15: Interleukin 15; CLEC4D: C-type lectin domain family 4 member D; IL1RN: Interleukin 1 receptor antagonist; ADAMTS13: A disintegrin and metalloproteinase with thrombospondin type 1 motif 13; APEX1: Apurinic/apyrimidinic endodeoxyribonuclease 1; CPED1: Cadherin like and PC-esterase domain containing 1; CRTAC1: Cartilage acidic protein 1; DFFA: DNA fragmentation factor subunit alpha; EGLN1: Egl-9 family hypoxia inducible factor 1; PDCD5: Programed cell death 5; FKBP4: FK506 binding protein 4; PSMG3: Proteasome assembly chaperone 3; RILP: Rab interacting lysosomal protein; EGFR: Epidermal growth factor receptor; BID: BH3 interacting domain death agonist; LGALS3: Galectin 3; HDGF: Hepatoma derived growth factor; OMG: Oligodendrocyte myelin glycoprotein; CEACAM8: Carcinoembryonic antigen related cell adhesion molecule 8; OLR1: Oxidized low density lipoprotein receptor 1;PGLYRP1: Peptidoglycan recognition protein 1; PARP1: Poly (ADP-ribose) polymerase 1;HGF: Hepatocyte growth factor; CCL11: C–C motif chemokine ligand 11; PTS: 6-Pyruvoyl tetrahydropterin synthase; CAPG: Capping actin protein, gelsolin like; PSIP1: PC4 and SFRS1 interacting protein 1; CSTB: Cystatin B; CCL2: C–C motif chemokine ligand 2; FABP5: Fatty acid binding protein 5; GLO1: Glyoxalase I;FUS: FUS RNA binding protein; ELOA: Elongin A; QDPR: Quinoid dihydropteridine reductase; ZBTB17: Zinc finger and BTB domain containing 17; TFF1: Trefoil factor 1; CCL20: C–C motif chemokine ligand 20; MMP7: Matrix metallopeptidase 7; METAP2: Methionyl aminopeptidase 2; AARSD1: Alanyl-tRNA synthetase domain containing 1; OGFR: Opioid growth factor receptor; SIT1: Signaling threshold regulating transmembrane adaptor 1; EIF4B: Eukaryotic translation initiation factor 4B; EIF4EBP1: Eukaryotic translation initiation factor 4E binding protein 1; HSPA1A: Heat shock protein family A (Hsp70) member 1A; DNAJB1: DNAJ heat shock protein family (Hsp40) member B1; PRDX5: Peroxiredoxin 5; TMSB10: Thymosin beta 10; LACTB2: Lactamase beta 2; BRK1: BRICK1 subunit Of SCAR/WAVE actin nucleating complex; IPCEF1: Interaction protein for cytohesin exchange factors 1; NUCB2: Nucleobindin 2; CASP8: Caspase 8; FKBP5: FK506 binding protein 5; MOG: Myelin oligodendrocyte glycoprotein; PTPRN2: Protein tyrosine phosphatase receptor type N2; KLK6: Kallikrein related peptidase 6; EGFL7: Epidermal growth factor like domain multiple 7; SCG2: Secretogranin II.

These three subsets of shared proteins were further analyzed using the med4way method to construct mediation models, revealing the relationship between individual air pollution factors and depression and anxiety. Detailed results of these models are presented in Supporting Information 10: Table S3a (four-way decomposition for PHQ2, subset 1), Supporting Information 11: Table S3b (four-way decomposition for all-cause depression, subset 2), and Supporting Information 12: Table S4a (four-way decomposition for GAD2, subset 3). For mediation models demonstrating consistent mediation effects, relationships are visually represented in Figure 3 using Sankey diagrams. Overall, a total of 61 unique proteins statistically demonstrated mediation in the association between air pollution and the occurrence of depression and depression/anxiety symptoms. We observed that PM_2.5_ affected the highest number of proteins contributing to anxiety and depression (39 proteins), followed by PM_10_ (21 proteins), NO_2_ (17 proteins), NO_x_ (18 proteins respectively), and PM_coarse_ (three proteins). Among the proteins mediating the effect of air pollution, nine were associated with Baseline PHQ2, 23 with Incident ICD9/10 Depression, and 38 with Baseline GAD2. The three proteins (LRRN1, Interleukin (IL)−15, C-type lectin (CLEC)-4D) showing relatively more associations were associated with at least three pollutants and linked to at least two outcomes.

**Figure 4:**
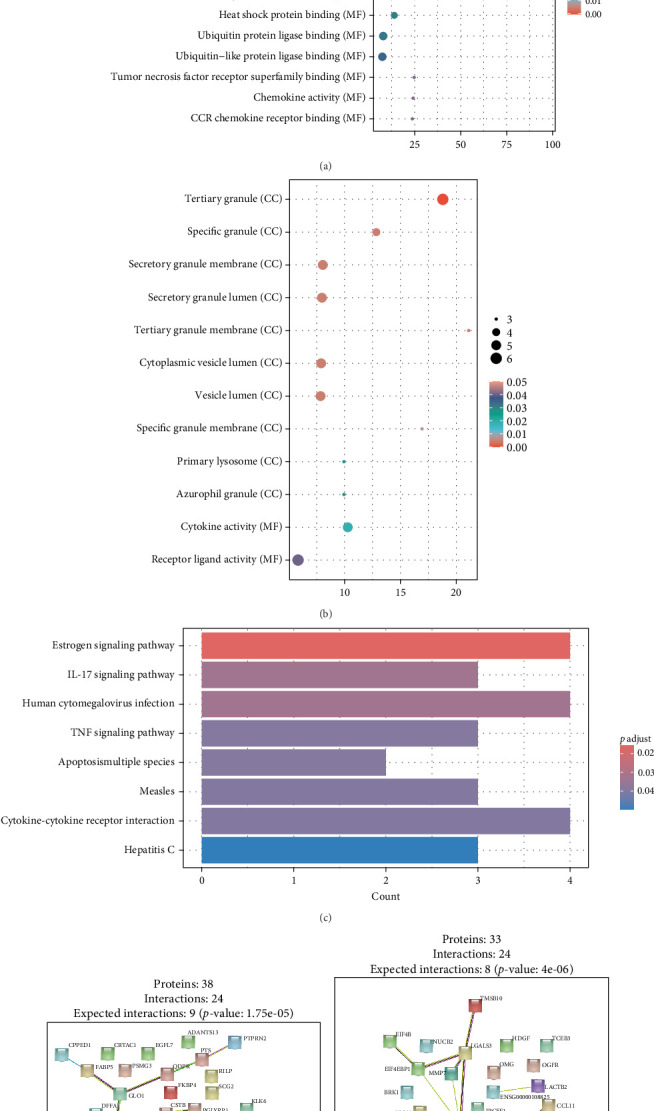
Bioinformatics Analysis Results. (A) GO analysis for mediating-effect proteins on air pollutants and depression. This figure showcases the GO analysis results, where the color of each point represents the adjusted *p*-value (*p* adjust), indicating the level of significance. The color gradient ranges from 0 to 0.05. The size of each point (size = Count) represents the number of genes associated with each GO term, reflecting the frequency of appearance. The horizontal axis indicates the fold enrichment associated with depression. The vertical axis shows the three GO ontologies - biological process (BP), molecular function (MF), and cellular component (CC), with the top 10 terms displayed for each. (B) GO analysis for mediating-effect proteins on air pollutants and anxiety. Similar to Figure 4A, this figure presents the GO analysis results for mediating-effect proteins related to anxiety. (C) KEGG enrichment analysis for mediating-effect proteins on air pollutants and depression. This figure displays the KEGG pathway enrichment analysis, where the horizontal axis (count) represents the number of genes associated with each KEGG pathway, and the vertical axis shows specific enriched KEGG pathways in the gene list. The color gradient of the bars indicates the adjusted *p*-value (*p* adjust), reflecting the significance level of each pathway. (D) Protein–protein interaction (PPI) network for mediating-effect proteins on air pollutants and depression. (E) Protein–protein interaction (PPI) network for mediating-effect proteins on air pollutants and anxiety.

Finally, we partitioned a total of 61 proteins into two categories for bioinformatics analysis, one associated with depression and the other with anxiety.  Figure 4A, B illustrate statistically significant GO terms for each category. Only the proteins related to depression showed statistically significant results in the KEGG pathway enrichment analysis (Figure 4C). In the PPI network analysis, despite both networks displaying low average local clustering coefficients (both 0.32), their PPI enrichment *p*-Values were both <0.001, indicating that these interactions are likely biologically meaningful (Figure 4D, E).

## 4. Supplementary Material Description

The process of identifying proteins that mediate the effects of air pollution on depression and anxiety in this study is multi-staged. Each stage of this process yields results that are documented in the supplementary materials. Throughout the analysis, proteins are only considered for further investigation if they pass multiple comparison correction using the FDR. The main text primarily showcases those mediating proteins that exhibit statistically significant associations.

## 5. Discussion

In this prospective study, we elucidated the protein-mediated effects of five major air pollutants on depression and anxiety, which are among the most prevalent mental disorders. Our findings revealed 23 mediating proteins associated with the risk of developing MDD due to air pollution, while no mediating proteins were identified for the risk of developing AD. Additionally, we detected 38 mediating proteins linked to air pollution-induced anxiety and 9 proteins associated with depressive symptoms. To our knowledge, this is one of the largest population-based cohort studies providing proteomic evidence on the mediating protein associations between air pollution and MDD/AD in older adults. GO and KEGG pathway analyses highlighted a distinct set of genes intricately involved in various biological processes and pathways, including Cellular Component categories such as tertiary granule, specific granule, and secretory granule membrane, as well as Molecular Function categories like cytokine activity and receptor ligand activity. Moreover, the KEGG pathway analysis identified significant associations with the Estrogen signaling pathway, IL-17 signaling pathway, and TNF signaling pathway. STRING analysis further underscored the shared roles of proteins IL15, C–C Motif Chemokine Ligand (CCL)−2 and CCL20 in the context of air pollution-induced depression and anxiety, potentially highlighting EGFR's critical role in depression. These findings suggest that the immune system plays a significant role in the biological mechanisms linking air pollution to depression and anxiety.

Our findings align with the expanding evidence that long-term exposure to air pollutants is associated with a heightened risk of MDDs, depressive symptoms, and anxiety. One study based on data from the UK Biobank cohort, which includes 389,185 participants, has elucidated significant associations between prolonged exposure to PM_2.5_, NO_2_, and NO_x_ and an increased risk of MDDs [5]. Another UKB cohort study, which employed the PHQ-4 as an outcome measure for depressive and anxious symptoms, further demonstrated that long-term exposure to PM_2.5_, NO_2_, NO, PM_coarse_ and PM_10_ is significantly associated with increased depressive and anxiety symptoms, with PM_2.5_ exhibiting the strongest correlation [9]. Our findings support these conclusions. In our research, we also explored potential mediating proteins implicated in the pathway between individual air pollution exposure and the onset of MDDs. Notably, our analysis identified several proteins (CLEC4D, LRRN1 and IL-15) that mediate the effects of PM_2.5_, NO_x_, and NO_2_ in relation to MDDs. Moreover, we did not observe any statistically significant mediation through proteins associated with PM_coarse_ and PM_10_ exposure in our study population. Furthermore, by incorporating the Air Pollution Index as an independent variable in our mediation analysis, we aimed to elucidate the overall pathways through which air pollutants influence depressive and anxiety symptoms. Using this approach, we did not find that the Air Pollution Index identified specific proteins as statistically significant mediators. This may also indicate that the differential impact of various pollutants on mediating proteins underscores the complexity of the mechanisms underlying air pollution-related mental health effects. Researchers will need to develop more sophisticated methods to study this issue comprehensively.

We identified and analyzed a set of proteins that play crucial roles in oxidative stress and inflammatory responses within cells. These proteins potentially mediate the link between air pollution and the incidence of depression. The proteins identified include IL1RN, BH3 interacting domain death agonist (BID), Galectin-3, CLEC4D, hepatoma-derived growth factor (HDGF), Oligodendrocyte Myelin Glycoprotein (OMG), Methionine aminopeptidase 2 (METAP2), Alanyl-tRNA editing protein (AARSD1), Opioid Growth Factor Receptor (OGFR), Signaling Threshold Regulating Transmembrane Adaptor 1 (SIT1), Eukaryotic Translation Initiation Factor 4B (EIF4B), Eukaryotic Translation Initiation Factor 4E Binding Protein 1 (EIF4EBP1), Heat shock 70 kDa protein 1 (HSPA1A), DnaJ Heat Shock Protein Family (Hsp40) Member B1 (DNAJB1), peroxidase 5 (PRDX5), Thymosin Beta 10 (TMSB10), Lactamase Beta 2 (LACTB2), Elongin A (ELOA), BRICK1 Subunit Of SCAR/WAVE Actin Nucleating Complex (BRK1), Interaction Protein For Cytohesin Exchange Factors 1 (IPCEF1), Nucleobindin 2 (NUCB2), Caspase 8 (CASP8), and FKBP Prolyl Isomerase 5 (FKBP5). Chemical substances and PM in air pollution can induce oxidative stress within cells through various pathways, including peroxidases like PRDX5 [37, 38] and factors regulating protein synthesis such as EIF4B and EIF4EBP1. These responses may lead to increased oxidative stress within cells, damaging cell membranes and nucleic acids, consequently affecting normal neural function. Additionally, air pollution can activate inflammatory response pathways (e.g., IL1RN [39], galectin-3 [40], CLEC4D [41], CASP8 [42, 43]), disrupting neurotransmitter balance and exacerbating neuronal inflammation, thereby increasing the incidence of depression [44, 45]. These proteins may regulate stress responses and protective mechanisms in neurons, and their abnormal expression or activity might contribute to the pathophysiological changes observed in patients with depression. Our study highlights the significant roles these proteins play in oxidative stress and inflammatory responses induced by air pollution, providing potential molecular mechanisms explaining how air pollution may increase depression risk. Our findings offer evidence from human population studies and are further supported by animal research. Moreover, the discovery of various proteins such as FKBP5 [45] and HSPA1A [46] reveals their involvement in cellular stress responses within neurons. METAP2 [47] may be implicated in regulating neuronal metabolic pathways, influencing neuronal survival. Understanding these proteins, including their functions in stress and oxidative protection (DNAJB1 [48], TMSB10), appetite regulation (NUCB2 [49] enhances our comprehension of how air pollution impacts the nervous system. This research may underscore the intricate mechanisms through which air pollution may affect neuronal function and overall neurological health.

Notably, we propose that IL-15 may play a crucial role in mediating the relationship between air pollutants and depressive mood and anxiety [50]. Previous research has indicated that increased levels of IL-5 [51], IL-6 [52], and IL-8 [42] are associated with heightened air pollution, potentially contributing to asthma, cardiovascular diseases, and metabolic risks. Our research found the significant role of IL-15 in the development of depressive mood induced by these environmental factors. PPI analysis has revealed that IL-15 may act as a pivotal factor in neuro-immuno-endocrine regulation [53, 54], integrating cellular responses triggered by various pollutants and serving as a key mediator in the development of depressive mood. Furthermore, studies have shown that IL-15 can promote localized inflammation within the nervous system. Pollutants such as NO_2_, NO_x_, PM_2.5_, and PM_coarse_ may induce similar inflammatory responses, impacting neuronal function and emotional regulation. IL-15, as an essential regulatory molecule, participates in this modulation process. These pollutants may also lead to cellular oxidative stress, with IL-15 playing a role in the regulation of the antioxidant defense system, maintaining the redox balance within neurons [55]. Additionally, IL-15 is involved in the regulation of synaptic development and plasticity [55]. Disruption of IL-15 function by pollutants may affect neuronal connectivity remodeling, consequently influencing emotion-related neural circuit activities [50]. Lastly, IL-15 can activate regulatory T cells and natural killer cells [56], further impacting neuronal function and emotional regulation. The imbalance of these immune cells may also be a mechanism by which pollutants induce depressive mood.

EGF plays a critical role in stimulating cell proliferation, growth, and differentiation through its interaction with the EGFR. Although EGFR is expressed in both the central and peripheral nervous systems, its neurotrophic functions, particularly within the central nervous system, have been underestimated and remain poorly investigate [57]. Recent studies have suggested that elevated levels of EGF may serve as a candidate biomarker for mood disorders, highlighting its potential relevance in psychiatric conditions [49]. Moreover, air pollution, particularly PM_2.5_, is known to accumulate in the alveoli, leading to inflammation and tissue damage [58]. The activation of EGFR is associated with PM_2.5_-induced pro-inflammatory responses, which can lead to neuroinflammation [59]. Furthermore, this neuroinflammation may disrupt the regulation of neurotrophic factors, such as brain-derived neurotrophic factor (BDNF) and EGF, both of which play critical roles in the pathophysiology of MDD [60, 61]. Therefore, the activation of EGFR not only triggers neuroinflammation but may also exacerbate the pathological processes of MDD by affecting the balance of neurotrophic factors [62]. Understanding the intricate relationship between EGFR activation, neuroinflammation, and the modulation of neurotrophic factors may provide valuable insights into the underlying mechanisms of mood disorders.

Our research suggests that the concurrent upregulation of CCL2 and CCL20 in response to air pollution may have a synergistic effect in promoting neuroinflammation, thereby mediating the relationship between air pollution and depression and anxiety. CCL2, known as monocyte chemoattractant protein-1 (MCP-1), is a crucial chemokine that facilitates the recruitment of monocytes, memory T cells, and dendritic cells to sites of inflammation. Exposure to air pollutants, particularly PM, has been shown to increase the expression of CCL2 in both peripheral and central nervous systems. Elevated levels of CCL2 can promote the infiltration of immune cells into the brain, leading to neuroinflammation, which is a significant risk factor for the development of depressive symptoms. Studies have demonstrated that CCL2 can disrupt the blood-brain barrier [63]facilitating the entry of peripheral inflammatory cells into the brain. This infiltration can activate microglia, the resident immune cells of the central nervous system, leading to the release of pro-inflammatory cytokines and further exacerbation of neuroinflammation. Such inflammatory responses have been implicated in synaptic plasticity deficits and altered neurotransmitter systems, which are critical in the pathophysiology of depression [64] and anxiety [65]. CCL20, known as macrophage inflammatory protein-3*α* (MIP-3*α*), plays a pivotal role in immune responses by attracting lymphocytes and dendritic cells. Similar to CCL2, air pollution exposure has been associated with increased CCL20 expression. Elevated CCL20 levels can lead to the activation of immune cells and subsequent release of inflammatory mediators within the central nervous system. This chemokine–receptor interaction has been shown to activate signaling pathways that contribute to neuroinflammation and neurodegeneration. Moreover, chronic neuroinflammation can affect neurogenesis and synaptic function, both of which are crucial for mood regulation and cognitive function.

In our study, we did not identify any mediator proteins associated with the onset of AD related to long-term exposure to air pollution. However, we did identify several mediator proteins linked to the development of depression resulting from prolonged exposure to air pollution. Additionally, we identified some mediator proteins that may mediate the impact of air pollution on both depressive and anxiety symptoms. In the pathophysiology of anxiety and depression, research has focused on the roles that cytokines, hormones, oxidative stress markers, and neuropeptides play [66]. This finding could indicate that AD is related to their transient nature, while depression may be associated with enduring changes in its pathology [2, 67]. The symptoms of anxiety frequently manifest in specific environments and contexts, making them more susceptible to immediate influence. However, there isn't much research on ADs' biomarkers [67, 68]. As a result, anxiety symptoms demonstrate heightened sensitivity to transient environmental fluctuations, such as air pollution, when examining serum-related protein-mediated pathways [69]. In contrast, the pathogenesis of depression typically involves more complex and long-term physiological changes, typically including neurotransmitter imbalances and sustained inflammatory responses [6]. The methodological approach used to assess stress symptoms may impact the discovered differences in our results. In particular, the diagnostic criteria for ADs were based on ICD standards, whereas the GAD scale was used to assess anxiety symptoms. The discovered variations in the results may have been caused by this gap. Future studies may look at the possible effects of various analysis tools on study outcomes and seek to further investigate the biological mechanisms that underlie AD.

Our research has notable strengths as well as certain limitations. Firstly, it is distinguished by being a thorough proteomic analysis conducted on a large cohort, making it the first study of its kind with sufficient statistical power to investigate how plasma proteomics mediate and moderate the relationship between air pollution and depression and anxiety. Secondly, the use of interconnected data, detailed diagnostic information, and careful, repeated evaluations of mental health status allows for the generation of multiple outcome variables. Lastly, the diverse participant pool in the UK Biobank enables an unbiased assessment of exposure–outcome connections by adjusting for potential confounding factors. However, several limitations must be noted when interpreting the results. Firstly, since the UK Biobank consists of a volunteer cohort, its participants may be healthier than the general population. This healthier status could potentially reduce the observed effects of air pollution, as these individuals might be less susceptible to psychosocial stress. Additionally, there is an acknowledged measurement bias in air pollution data. Our air pollution measurements primarily reflect the average outdoor air pollution levels for the year 2010, with no further measurements in subsequent studies. Baseline visits for the UK Biobank occurred between 2006 and 2010, which limits our ability to explore the lagged or short-term effects of air pollution on depression and anxiety. Furthermore, considering that individuals, particularly older adults, spend a significant portion of their time indoors, personal exposure to air pollutants may not directly correspond to the outdoor environmental concentrations that were analyzed in this study [70]. Although the study accounted for various confounding factors, the nature of observational research means residual confounding is inevitable. The use of four-way decomposition models is based on strong assumptions, particularly the absence of unmeasured confounding factors, which cannot be conclusively ruled out, nor can experimental evaluation be conducted. Furthermore, we cannot exclude the possibility of exposure-induced mediation-outcome confounding. To our knowledge, the med4way approach does not incorporate innovative techniques to reduce this potential confounding. Additionally, although cases of depression and anxiety were excluded, reverse causation remains a possible explanation [21]. The UK Biobank may not be representative of the general UK population, and our analysis lacked sufficient statistical power to stratify by ethnicity, gender, or age groups. Moreover, only about one-third of participants completed the 7-year follow-up survey, which may limit the study's power to detect statistically significant mediating proteins at extended time points. This subgroup also had a higher proportion of individuals with MDD and/or AD compared to those without these conditions at baseline, suggesting that the real-world burden of MDD and/or AD during follow-up might be worse than indicated by current information. Lastly, as with most studies, the onset of MDD and/or AD remains unknown. There may also be misclassification and underestimation of newly developed depression and anxiety outcomes.

In conclusion, our study represents the largest investigation to date utilizing proteomic data to explore the potential mediating mechanisms underlying the associations between long-term exposure to major air pollutants and the risks of depression and anxiety. Our results reveal that the link between depression and air pollution can be mediated by a variety of plasma proteins, but we did not identify plasma proteins that mediate the association between air pollution and the occurrence of anxiety. This suggests that depression and anxiety may involve distinct biological mechanisms. Future research should further explore the relevant biological features associated with air pollution and the risk of depression and anxiety. This study provides important insights into the mechanisms by which environmental factors influence mental health, and offers potential biomarkers for the prevention and treatment of depression and anxiety-related disorders.

## Figures and Tables

**Table 1:** Population Characteristics in three cohorts.

Characteristic	Basic cohort	Survival cohort	MDD/AD cohort
*n*	43,169	38,301	4868
Age (mean (SD))	56.81 (8.19)	56.93 (8.22)	55.88 (7.90)
Age group (%)			
Less than 45	8939 (20.7)	8202 (21.4)	737 (15.1)
45 to 49	5393 (12.5)	4708 (12.3)	685 (14.1)
50 to 54	6207 (14.4)	5438 (14.2)	769 (15.8)
55 to 59	7654 (17.7)	6654 (17.4)	1000 (20.5)
60 to 64	10,517 (24.4)	9369 (24.5)	1148 (23.6)
Greater than 64	4459 (10.3)	3930 (10.3)	529 (10.9)
Sex (%)			
Female	23,108 (53.5)	19,989 (52.2)	3119 (64.1)
Male	20,061 (46.5)	18,312 (47.8)	1749 (35.9)
Race and ethnicity (%)			
Asian	775 (1.8)	711 (1.9)	64 (1.3)
Black	910 (2.1)	861 (2.2)	49 (1.0)
Multiracial	289 (0.7)	253 (0.7)	36 (0.7)
Other	554 (1.3)	491 (1.3)	63 (1.3)
White	40,641 (94.1)	35,985 (94.0)	4656 (95.6)
Townsend deprivation index (mean (SD))	−1.32 (3.08)	−1.38 (3.05)	−0.87 (3.27)
Educational qualifications (%)			
College or university degree	14,140 (32.8)	12,709 (33.2)	1431 (29.4)
Other	29,029 (67.2)	25,592 (66.8)	3437 (70.6)
Employment (%)			
Active	23,955 (55.5)	21,569 (56.3)	2386 (49.0)
Inactive	19,214 (44.5)	16,732 (43.7)	2482 (51.0)
Length of time at residence (mean (SD))	17.51 (12.15)	17.69 (12.15)	16.13 (12.04)
Household income (%)			
Less than 18,000	9653 (22.4)	8117 (21.2)	1536 (31.6)
18,000 to 30,999	12,068 (28.0)	10,710 (28.0)	1358 (27.9)
31,000 to 51,999	11,592 (26.9)	10,375 (27.1)	1217 (25.0)
52,000 to 100,000	7854 (18.2)	7228 (18.9)	626 (12.9)
Greater than 100,000	2002 (4.6)	1871 (4.9)	131 (2.7)
Healthy alcohol intake status (%)			
Yes	25,239 (58.5)	22,454 (58.6)	22,454 (58.6)
No	11,953 (27.7)	10,679 (27.9)	1274 (26.2)
Healthy physical activity status (%)			
Yes	27,666 (80.7)	24,803 (81.4)	2863 (75.7)
No	6600 (19.3)	5682 (18.6)	918 (24.3)
Urban residency (%)			
Yes	36,545 (84.7)	32,383 (84.6)	4162 (85.5)
No	6202 (14.4)	5555 (14.5)	647 (13.3)
FEV1	2.72 (0.79)	2.73 (0.80)	2.61 (0.76)
FVC	3.60 (1.03)	3.62 (1.04)	3.45 (0.98)
Diabetes (%)			
Yes	2395 (5.5)	2092 (5.5)	2092 (5.5)
No	40,765 (94.5)	36,200 (94.5)	36,200 (94.5)
Hypertension (%)			
Yes	23,893 (55.4)	21,261 (55.5)	21,261 (55.5)
No	19,267 (44.6)	17,031 (44.5)	17,031 (44.5)
Coronary heart disease (%)			
Yes	2381 (5.5)	2022 (5.3)	2022 (5.3)
No	40,779 (94.5)	36,270 (94.7)	36,270 (94.7)
Proximity to major roads (median [IQR])	2.65 [1.31, 6.04]	2.64 [1.31, 6.02]	2.72 [1.36, 6.30]
24 h weighted average noise (median [IQR])	54.90 [53.44, 57.04]	54.90 [53.45, 57.04]	54.90 [53.39, 57.02]
Greenspace buffered at 1000 m (mean (SD))	44.87 (21.16)	44.91 (21.25)	44.58 (20.37)
BMI (mean (SD))	27.43 (4.76)	27.35 (4.69)	28.04 (5.23)
NO2 (median [IQR])	26.20 [21.33, 31.29]	26.16 [21.29, 31.30]	26.46 [21.70, 31.25]
NOx (median [IQR])	42.32 [34.25, 50.82]	42.25 [34.14, 50.76]	42.89 [35.09, 51.30]
PM10 (mean (SD))	16.23 (1.90)	16.23 (1.91)	16.25 (1.88)
PM2.5 (mean (SD))	10.00 (1.07)	9.99 (1.06)	10.08 (1.08)
PMcoarse (median [IQR])	6.12 [5.85, 6.65]	6.12 [5.85, 6.66]	6.12 [5.85, 6.60]
PHQ-4 score (mean (SD))	1.64 (2.13)	1.42 (1.88)	3.35 (3.07)
GAD-2 (mean (SD))	1.04 (1.24)	0.94 (1.14)	1.90 (1.63)
PHQ-2 (mean (SD))	0.60 (1.13)	0.49 (0.98)	1.45 (1.70)

## Data Availability

The datasets analyzed in this study are available through the UK Biobank (Application Number 104685). Researchers can apply for data access via the UK Biobank website (https://www.ukbiobank.ac.uk). Access requires approval of a research proposal and payment of associated administrative fees, as stipulated by the UK Biobank governance framework.The R 4.3 and STATA 18 code used for the statistical analysis in this study is available upon request from WM.

## Nomenclature

AARSD1: Alanyl-tRNA editing protein
AD: Anxiety disorder
ADAMTS13: A disintegrin and metalloproteinase with thrombospondin motifs 13
BID: BH3 interacting domain death agonist
BRK1: BRICK1 subunit of SCAR/WAVE actin nucleating complex
CASP8: Caspase 8
CCL: C–C motif chemokine ligand
CDE: Controlled direct effect
CHD: Coronary heart disease
CLEC: C-type lectin
DNAJB1: DnaJ heat shock protein family (Hsp40) member B1
EIF4B: Eukaryotic translation initiation factor 4B
EIF4EBP1: Eukaryotic translation initiation factor 4E binding protein 1
EGF: Epidermal growth factor
EGFR: Epidermal growth factor receptor
ELOA: Elongin A
FDR: False discovery rate
FKBP5: FKBP prolyl isomerase 5
GAD: Generalized Anxiety Disorder Scale
GO: Gene ontology
HDGF: Hepatoma-derived growth factor
HSPA1A: Heat shock 70 kDa protein 1
ICD-9/10: International Classification of Diseases-9/10
IL: Interleukin
IPCEF1: Interaction protein for cytohesin exchange factors 1
KEGG: Kyoto encyclopedia of genes and genomes
LACTB2: Lactamase beta 2
LRRN1: Leucine-rich repeat neuronal protein 1
MCP-1: Monocyte chemoattractant protein-1
MDD: Major depressive disorder
METAP2: Methionine aminopeptidase 2
MIP-3α: Macrophage inflammatory protein-3 alpha
NOx: Nitrogen oxides
NUCB2: Nucleobindin 2
OGFR: Opioid growth factor receptor
OMG: Oligodendrocyte myelin glycoprotein
PHQ: Patient Health Questionnaire
PIE: Pure indirect effect
PM: Particulate matter
PPI: Protein-protein interaction
PRDX5: Peroxidase 5
SIT1: Signaling threshold regulating transmembrane adaptor 1
TE: Total effect
TMSB10: Thymosin beta 10.

## Supporting Information

Supporting Information 1 FigureS1 (A) MDD-related air pollution index and various pollutants correlation matrix. (B) AD-related air pollution index and various pollutants correlation matrix.

Supporting Information 2 TableS1a_Protein-air pollution index derived from depression multivariable regression analysis results.

Supporting Information 3 TableS1b_Baseline PHQ2-protein multivariable regression analysis results (1).

Supporting Information 4 TableS1c_Protein-Incident depression multivariable cox regression results.

Supporting Information 5 TableS1d_PHQ9 at 7y-protein multivariable regression analysis results.

Supporting Information 6 TableS2a_Protein-air pollution index derived from anxiety multivariable regression analysis results.

Supporting Information 7 TableS2b_Baseline GAD2-protein multivariable regression analysis results.

Supporting Information 8 TableS2c_Protein-incident anxiety multivariable cox regression results.

Supporting Information 9 TableS2d_GAD7 at 7y -protein multivariable regression analysis results.

Supporting Information 10 TableS3a_Four-way decomposition of the association between individual air pollution and PHQ2 through selected plasma proteomic biomarkers (*k* = 25).

Supporting Information 11 TableS3b_Four-way decomposition of the association between individual air pollution and all-cause depression through selected plasma proteomic biomarkers (*k* = 65).

Supporting Information 12 TableS4a_Four-way decomposition of the association between individual air pollution and GAD2 through selected plasma proteomic biomarkers (*k* = 72).
